# Supplementary figures and images for: Calcium transfer from the ER to other organelles for optimal signaling in Toxoplasma gondii
Source: eLife. 2025 Nov 12;13:RP101894. doi: 10.7554/eLife.101894 (PMC12611264; doi:10.7554/eLife.101894)

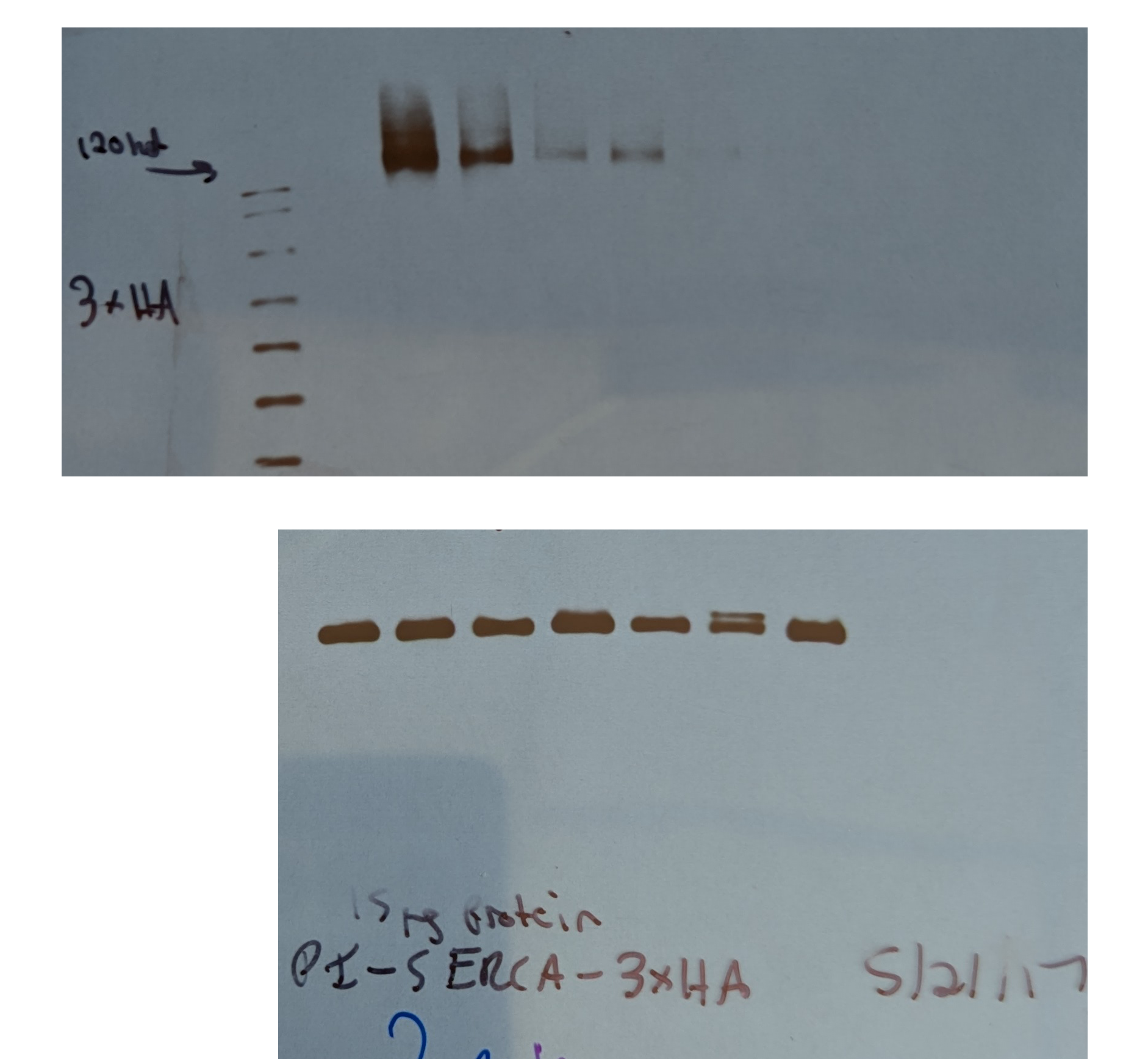

Supplement: Figure 3—source data 2. [file elife-101894-fig3-data2.zip › Figure 3-source data 2.tif]

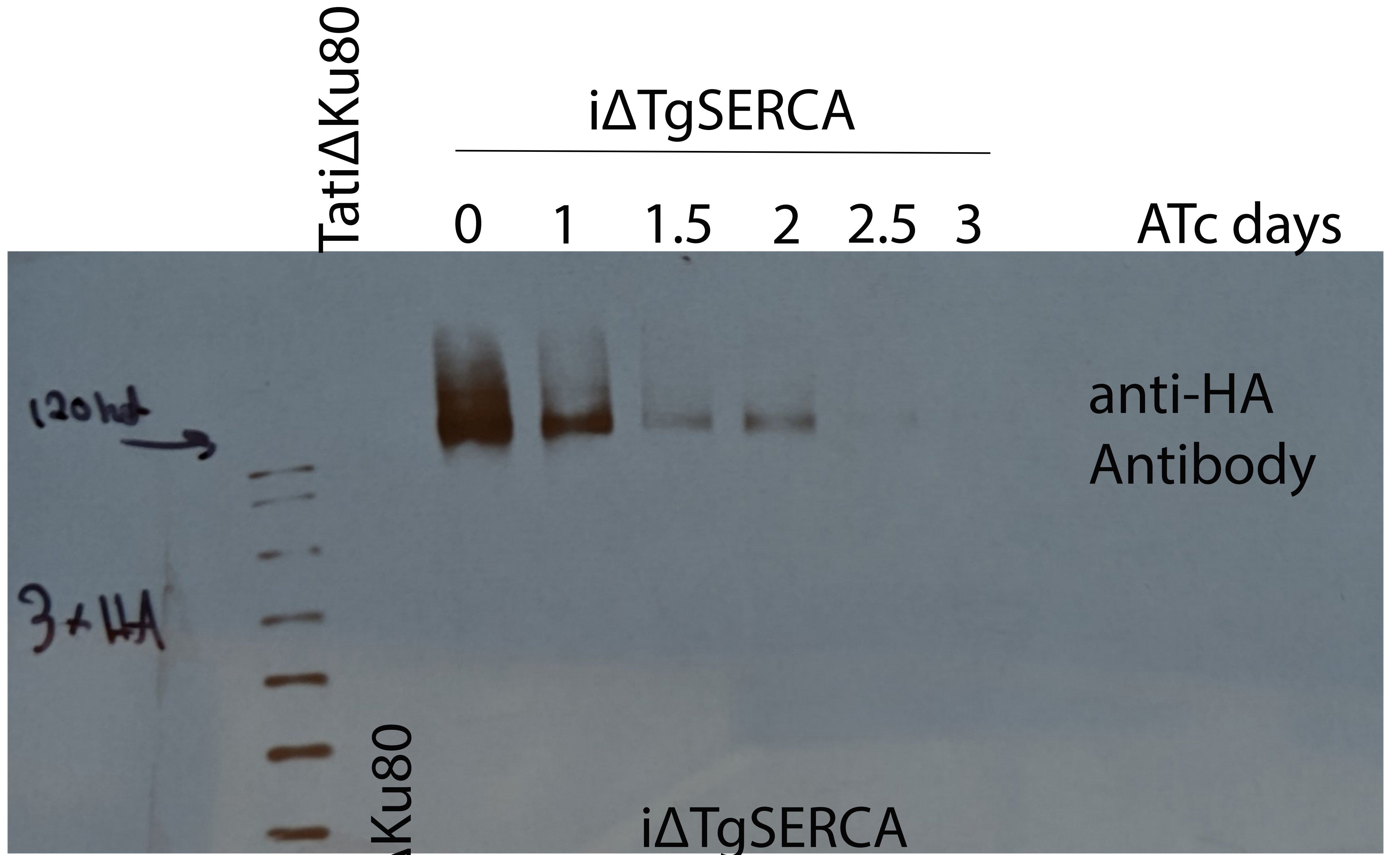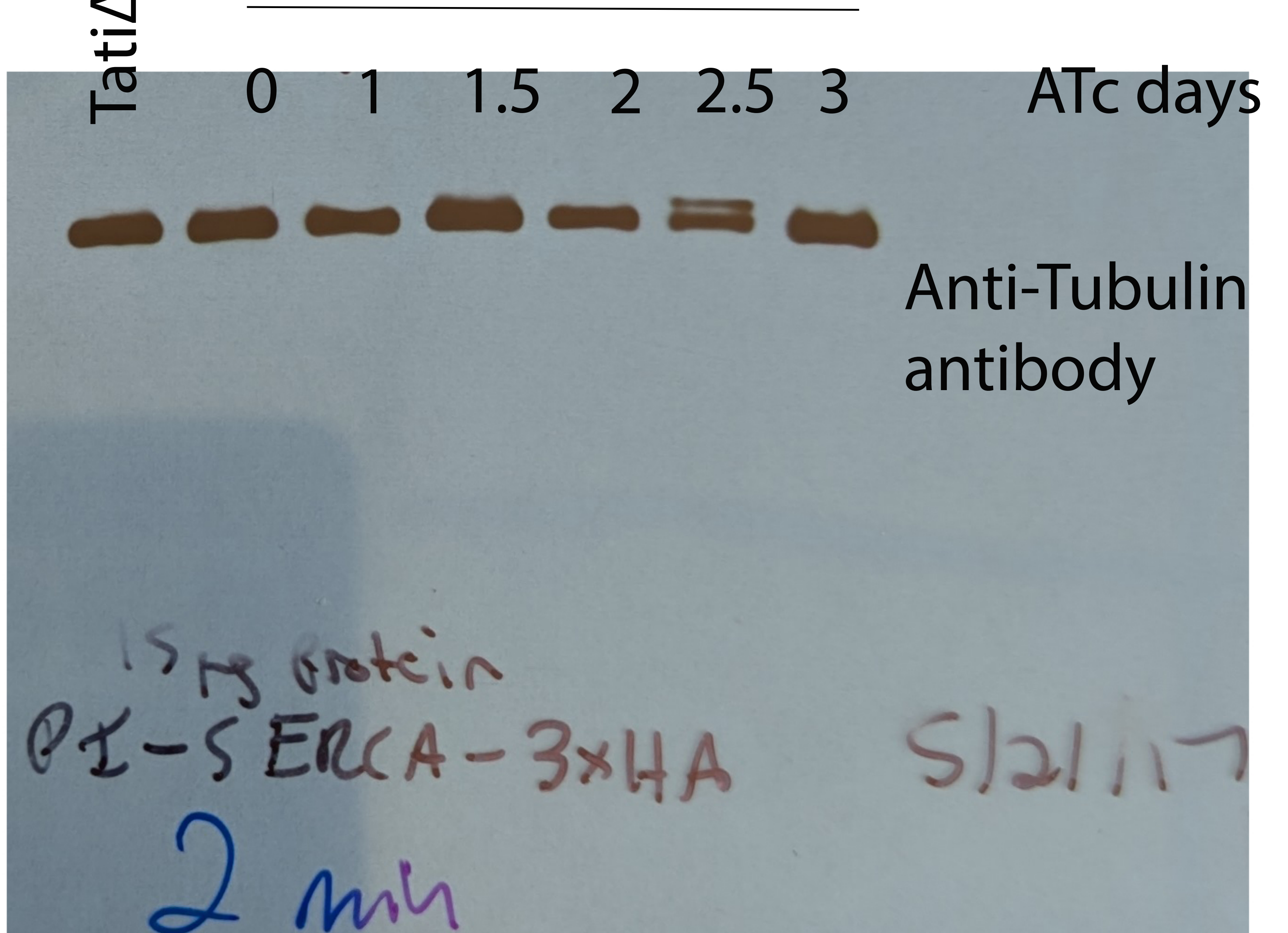

Supplement: Figure 3—source data 3. [file elife-101894-fig3-data3.zip › Figure 3-source data 3.pdf]

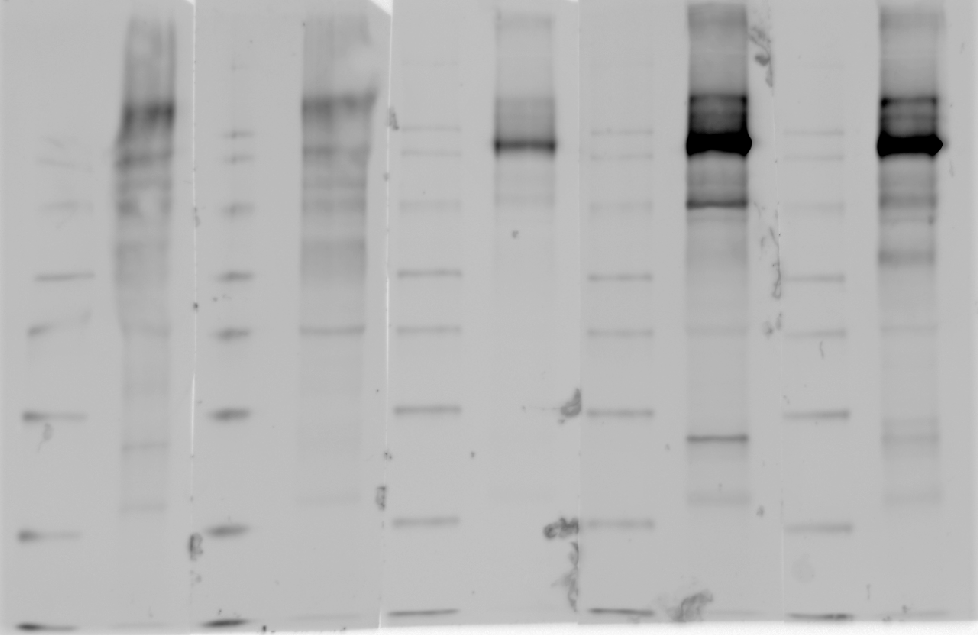

Supplement: Figure 3—figure supplement 1—source data 1. [file elife-101894-fig3-figsupp1-data1.zip › Figure 3-figure supplement 1-Source data 1.tif]

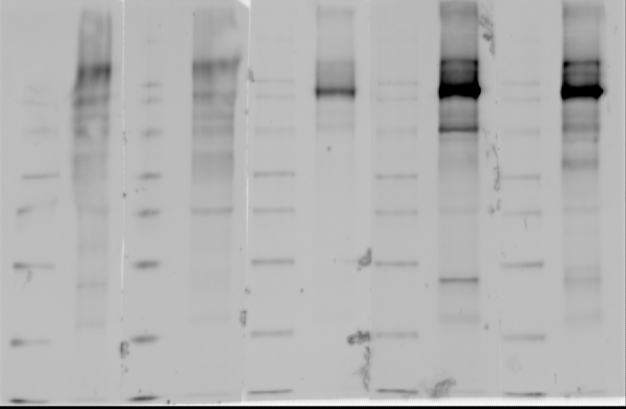

Marker

Sibley lab

anti-TgSERCAs

Marker

Guinea pig 2

anti-TgSERCAs

Supplement: Figure 3—figure supplement 1—source data 2. [file elife-101894-fig3-figsupp1-data2.zip › Figure 3-figure supplement 1-Source data 2.pdf]
