# Supplementary material for: Calcium transfer from the ER to other organelles for optimal signaling in Toxoplasma gondii
Source: eLife. 2025 Nov 12;13:RP101894. doi: 10.7554/eLife.101894 (PMC12611264; doi:10.7554/eLife.101894)
Supplement: Supplementary file 1. [file elife-101894-supp1.pdf]

Supplementary File 1: Primers used in this study

Primer Number	Sequence	Purpose
1: SERCA_5'UTR_F	ttctgcagatatccatcacactggcCCACCTCCTCGGCGGTTCTCTCTCG	Amplify 5'UTR of *TgSERCA*
2: SERCA_5'UTR_R	aggtttcgtgctgCGCTGCGTCTCCGAAGATAAGCCGAAC	
3: DHFR+T7S4_SERCA_F	cggagacgcagcgCAGCACGAAACCTTGCATTCAAACC	Amplify DHFR+T7S4 cassette
4: DHFR+T7S4_SERCA_R	ttgacaggtccatGGTTGAAGACAGACGAAAGCAGTTG	
5: SERCA_3'HR_F	tctgtcttcaaccATGGACCTGTCAAACGAGAAAGCCG	Amplify the 3' homologous region of *TgSERCA* for promoter insertion
6: SERCA_3'HR_R	gggccctctagatgcatgctcgagcGGAGACTCTGAATGAGTGAACACGA G	
7: SERCA-LIC-F	TACTTCCAATCCAATTTAATGCCGACGATCCCTGCTCCTT	Amplify 3' *TgSERCA* for LIC into plic-3HA
8: SERCA-LIC-R	TCCTCCACTTCCAATTTTAGCCTGCAGCTTGCGCAGCTG	
9: R-6f-AvrII-NoSC	CCTAGGCTTCGCTGTCATCATTTGTACA	Make SOD2-Gcamp6 construct
10: F-SOD2-BglII	AGATCTATGTCCATCACAGCTGTCCTAGTGCCAG	
11: R-SOD2-4-Gcamp6	TGAGAACCCATGGCGTTTGTGGAGAAACAGTGGGC	Make SOD2-Gcamp6 construct
12: F-Gcamp6-4-SOD2	CCACAAACGCCATGGGTTCTCATCATCATCATCATC	
13: XmaI_SERCA_PNP_F	5' ACGTCCCGGGTGCCATCGTGAGAAAGCTCGCG 3'	Amplify *TgSERCA* to prepare recombinant protein for Ab production
14: HindIII_SERCA_PNP_R:	5' ACGTAAGCTTGTTGTCGTCTGCGAGAACCATG 3'	
